# Reduced T Cell Receptor Excision Circle Levels in the Colonic Mucosa of Microscopic Colitis Patients Indicate Local Proliferation rather than Homing of Peripheral Lymphocytes to the Inflamed Mucosa

**DOI:** 10.1155/2013/408638

**Published:** 2013-07-14

**Authors:** Ashok Kumar Kumawat, Kristina Elgbratt, Curt Tysk, Johan Bohr, Elisabeth Hultgren Hörnquist

**Affiliations:** ^1^School of Health and Medical Sciences, Örebro University, 70182 Örebro, Sweden; ^2^Division of Gastroenterology, Department of Medicine, Örebro University Hospital, 70185 Örebro, Sweden; ^3^School of Medicine, Örebro University, 70182 Örebro, Sweden

## Abstract

Dysregulated T cell responses in the intestine may lead to chronic bowel inflammation such as collagenous colitis (CC) and lymphocytic colitis (LC), together known as microscopic colitis (MC). Having demonstrated increased local T cell responses in the intestinal mucosa of MC patients, we investigated the recent thymic emigrants by measuring T cell receptor excision circle (TREC) levels in the colonic biopsies from CC (*n* = 8), LC (*n* = 5), and CC or LC patients in histopathological remission (CC-HR, *n* = 3) (LC-HR, *n* = 6), non-inflamed diarrhoea patients (*n* = 17), and controls (*n* = 10) by real-time PCR. We observed lower median TREC levels in both CC and LC patients as well as in LC-HR patients compared to controls. In contrast to MC patients, non-inflamed diarrhoea patients presented with enhanced TREC levels compared to controls. None of the recorded differences did, however, reach statistical significance. A trend towards increased relative expression of CD3 was noted in all MC subgroups examined and reached statistical significance in LC patients compared to controls. In conclusion, reduced TRECs level in the colonic mucosa, together with our previously demonstrated enhanced expression of Ki67^+^ T cells, suggests local expansion of resident T lymphocytes in the inflamed mucosa of MC patients.

## 1. Introduction

Microscopic colitis (MC) is a chronic inflammatory bowel condition of unknown aetiology comprising collagenous colitis (CC) and lymphocytic colitis (LC) with an annual incidence each of 5-6 cases per 100.000 individuals [1–3]. Although the data on pathophysiology are still limited, it is postulated that MC is caused by disturbed immune responses to luminal antigen(s) in predisposed individuals [4]. 

It is generally appreciated that aberrant T cell responses may lead to chronic gut inflammatory conditions like inflammatory bowel disease [5]. Careful evaluation of thymic activity is of great interest since new naive T cells are made immunocompetent in thymus. Thymectomy has previously been demonstrated to prevent relapse in ulcerative colitis (UC) patients [6]. Our previous studies on G*α*i2^−/−^ spontaneous and dextran-sodium-sulphate- (DSS-) induced mouse models of colitis demonstrated aberrant thymocyte development characterized by enhanced numbers of mature and decreased number of immature thymocytes before and during onset of colitis, as well as decreased migratory responsiveness to intrathymic chemokines [7, 8]. 

The T cell receptor (TCR) consists of either of two types of heterodimers: *αβ*TCRs or *γδ*TCRs. Generation of T cell repertoires with diverse antigen specificities is achieved by random rearrangement of TCR gene segments (V-D-J, variable, diversity, and joining) in thymus [9, 10]. This process is initiated by recognition of recombination signal sequences (RSS) that flank the coding sequence and during this process the two signal ends are circularized, forming an extrachromosomal circular excision product [10]. These so-called T cell receptor excision circles—TRECs—are stable but are not replicated during mitosis and are consequently diluted with each cell division [11, 12]. Thus, TRECs levels are a direct reflection of the amount of recent thymic emigrants (RTE) in the periphery. TRECs measurements are used extensively to document T cell reconstitution following treatment of HIV infection and in patients who have undergone haematopoietic stem cell transplantation [9, 13, 14]. 

Increased mucosal mRNA expression of IFN-*γ* and TNF-*α* has been observed in the colonic mucosa of both CC and LC patients [15]. Recent flow cytometric analyses by our group have shown heavy infiltration of CD8^+^ intraepithelial lymphocytes (IELs) in the mucosa of CC and especially in LC patients [16]. Whether the heavy infiltration of CD8^+^ IELs is due to a larger influx of T cells, recent thymic emigrants, or an increased expansion of resident T cells in the mucosa of CC and LC patients, is still unknown. Such information is a first step toward understanding whether the activating antigen(s) resides in the mucosa or is rather transported via, for example, dendritic cells to the draining lymph nodes to activate naïve T cells there, thereby adding information on the nature of the chronic colonic inflammation in the mucosa of MC patients.

The aim of this study were to investigate the TREC levels in the CD3^+^ T cell compartment in the colonic mucosa of CC and LC patients compared to controls, using real-time PCR analysis for CD3 mRNA expression and real-time PCR analysis for TRECs analysis.

## 2. Material and Methods

### 2.1. Patients

**Figure 1:**
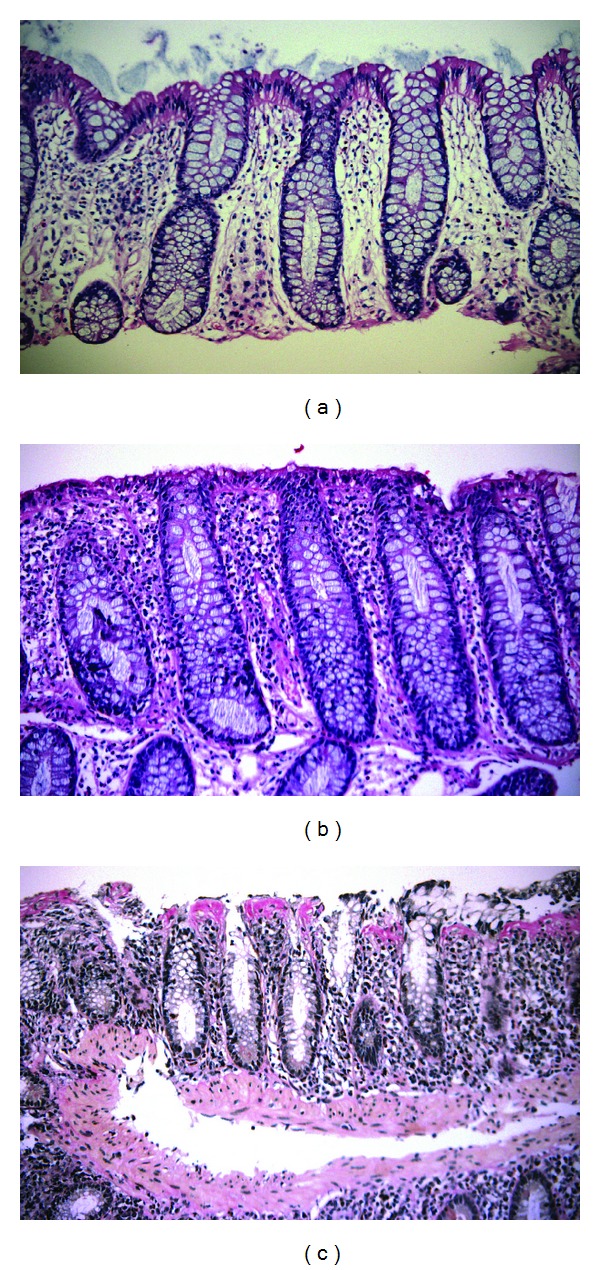
Human colonic biopsies showing (a) normal colonic mucosa; (b) typical findings of lymphocytic colitis, including epithelial cell damage with increased numbers of IELs, and infiltration of lymphocytes in the lamina propria; (c) typical findings of collagenous colitis, including increased thickness of the subepithelial collagen layer, inflammation of lamina propria, and epithelial cell damage with IELs. Photo: Sune Eriksson Department of Pathology, Örebro University Hospital. ((a) and (b)) H&E Staining, (c) Van Gieson Staining.

Clinically active LC and CC were defined as ≥3 loose or watery stools/day and/or abdominal pain. The diagnostic criteria for LC were histological findings (Figure 1(b)) of increased numbers of IELs (≥20/100 surface epithelial cells) in conjunction with surface epithelial cell damage and infiltration of lymphocytes in the lamina propria, but a normal collagen layer [3]. In CC, in addition to lymphocytic infiltration in the lamina propria and the epithelium, deposition of a subepithelial collagen layer of ≥10 *μ*m is seen (Figure 1(c)) [3]. 

MC patients fulfilling the above histopathological criteria and previously diagnosed with CC, LC, or UC and ongoing clinically active disease were recruited in the study. Exclusion criteria for both MC patients and diarrhoea controls were a previous history of Crohn's disease and/or clinical signs of gastrointestinal infection, ischemic colitis, or neoplastic disease. 

Colon biopsy specimens from 8 patients with CC, 5 with LC, and 4 with UC were investigated. In addition, we also identified a subgroup of MC patients who were previously diagnosed with LC (*n* = 6) or CC (*n* = 3), but who at the time of biopsy collection showed no histological signs of inflammation despite clinical symptoms of active LC or CC. Biopsies from these patients were grouped separately and are referred to as LC-histopathological remission (LC-HR) and CC-histopathological remission (CC-HR). The colonoscopic examination revealed that CC and LC patients generally had normal mucosa but occasionally with slight oedema or erythema. We also investigated biopsies from 17 patients with chronic, nonbloody diarrhoea, but with histologically normal mucosa and no earlier diagnosis of MC or IBD (*n* = 17) who are referred to as noninflamed diarrhoea patients. The noninflamed diarrhoea patients had an endoscopically normal mucosa, except for insignificant findings such as diverticulosis or polyps in some patients. Finally, 10 controls without diarrhoeal symptoms were recruited among patients undergoing colonoscopy for examination of gastrointestinal bleeding or abnormal radiological findings. All had normal findings at colonoscopy and histopathologic examination of colonic biopsies. None of the patients, irrespective of being in histological remission or having active disease, were treated with immunosuppressive drugs or antibiotics. The demographic features of the patients included in the study, as well as information on their use of nonsteroidal anti-inflammatory drugs (NSAIDs) and proton pump inhibitors (PPIs), are presented in Table 1.

Six biopsies from each individual were obtained using standard biopsy forceps, placed immediately in RNAlater (Ambion, Austin, TX, USA), and stored at −80°C until further processing. In MC patients and controls, they were taken from the right flexure, whereas in UC patients they were taken from macroscopically affected areas of the colon. The colonoscopies were performed at the division of Gastroenterology, Örebro University Hospital, Sweden, between March 2009 and October 2011. All patients in this study had provided written informed consent. The study was approved by the regional ethical committee of Örebro Uppsala County, Sweden (ID number 2008/278; 081015).

### 2.2. Total DNA/RNA Extraction, cDNA Synthesis, and Relative Quantification by Real-Time PCR

Total DNA and RNA were extracted from the same biopsy with AllPrep DNA/RNA Mini Kit (Qiagen, Hilden, Germany) according to the manufacturer's protocol. One *μ*g of total RNA was used for first-strand cDNA synthesis with random primers, using a high-capacity cDNA reverse transcriptase kit, according to the manufacturer's instructions (Applied Biosystems, Foster City, CA, USA). Both DNA and cDNA were stored at −80°C until further processing.

Real time PCR was performed using the thermal cycler TaqMan 7900 Fast Real-Time PCR System (Applied Biosystems) with 7900 Fast Sequence Detection and Relative Quantification software packages. Combined primers and probes for CD3*γ*, GAPDH, and TRECs were purchased from Applied Biosystems: CD3G: Hs00962186_m1 and GAPDH: Hs99999905_m1. The sequences for TRECs primers and probes were as follows: HuTRECs Forward; 5′-CCA TGC TGA CAC CTC TGG TT-3′, HuTRECs Reverse; 5′-TCG TGA GAA CGG TGA ATG AAG-3′; and HuTRECs Probe: FAM-5′-CAC GGT GAT GCA TAG GC ACCT GC-3′-TAMRA. PCR reactions were performed in 15 *μ*L volume using 1X TaqMan Universal PCR Master Mix (Applied Biosystems) and 100 ng cDNA for CD3*γ* and 100 ng DNA templates for TRECs analysis. For TRECs analysis, 15 *μ*L PCR reactions contained 11 *μ*M of each primer, 2.5 *μ*M of probe, 7.5 *μ*L of TaqMan Universal PCR Master Mix, and nuclease free water. PCR cycling conditions were as follows: Step one: 95°C for 2min, step two: 95°C for 3 s and 60°C for 30 s. The second step was repeated 45 times. Reactions were run in triplicates in a 96-well plate. Both DNA and mRNA were prepared from the same tissue and all the samples for CD3*γ* and TRECs assays were analyzed in the same plate to exclude assay to assay variability.

The relative values were calculated by the 2^(−ΔΔCt)^ method, where TREC expression levels were normalized against CD3*γ* mRNA, which was used as a reference for the total amount of T cells. CD3*γ* expression levels were normalized against the reference gene GAPDH and expressed as relative expression.

### 2.3. Statistical Analysis

Mann-Whitney two-tailed nonparametrical test was used for statistical comparison between groups, and data are presented with median values in the graphs. Differences were considered statistically significant when *P* ≤ 0.05. One patient in LC-HR group was defined as a statistical outlier (*P* < 0.05) according to Grubbs test, and therefore data from this patient was excluded while performing statistical comparison between groups. Spearman correlation analysis was performed to correlate TREC levels to age. Statistical analyses were performed using the GraphPad Prism 5 software for Windows. 

## 3. Results 

### 3.1. Reduced TREC Levels in the Mucosa of CC and LC Patients

**Figure 2:**
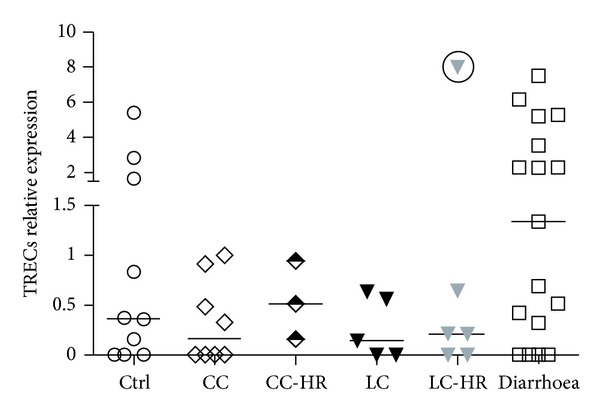
Levels of T cell receptor excision circles (TRECs) in colonic mucosa of patients with collagenous colitis (CC), CC-histopathological remission (CC-HR), lymphocytic colitis (LC), LC-histopathological remission (LC-HR), noninflamed diarrhoea patients and controls. The relative expression was calculated by the 2^(−ΔΔCt)^ method. Each symbol represents data from one individual. One symbol with the circle in LC-HR group represents a statistical outlier, and was excluded from the statistical calculations. Controls, *n* = 10; CC, *n* = 8; CC-HR, *n* = 3; LC, *n* = 5; LC-HR, *n* = 6; and noninflamed diarrhoea, *n* = 17.

We first investigated the TREC levels in the colonic biopsies relative to the amount of CD3^+^ T cells. The median TREC levels were lower in CC and LC as well as in LC-HR patients compared to controls (Figure 2). In fact, four out of eight of the CC patients, two out of five of the LC patients, and two out of six of the LC-HR patients (mean 42%) had undetectable level of TRECs compared to three out of ten in the control group (30%). However, the changes did not reach statistical significance. In contrast, CC patients in histological remission (CC-HR) did not have altered median TREC levels compared to controls despite clinical symptoms of active CC. Of the two CC patients who were treated with NSAIDs, one presented with the highest TREC levels within the group whereas the other patient had undetectable levels of TRECs, suggesting that NSAIDs did not affect the TREC levels in our study.

In contrast to the MC patients, but similar to our previous studies on isolated mucosal lymphocytes [17], increased levels of TREC were observed in UC patients, median (range) 0.6 (0.0–1.2) compared to controls. The noninflamed diarrhoea patients presented with very close to statistically significantly enhanced TRECs level (*P* = 0.054) compared to controls. No differences could be detected in median TREC levels in either CC-HR or LC-HR patients compared to active CC and LC patients.

Thymus size and activity decrease with increasing age. We therefore investigated the possible correlation between age and TRECs levels in the control group, where the age span was wider. However, we found no significant correlation (*P* = 0.32) between reduced TREC levels and increasing age (data not shown).

### 3.2. Upregulated CD3*γ* mRNA Levels in LC Patients

**Figure 3:**
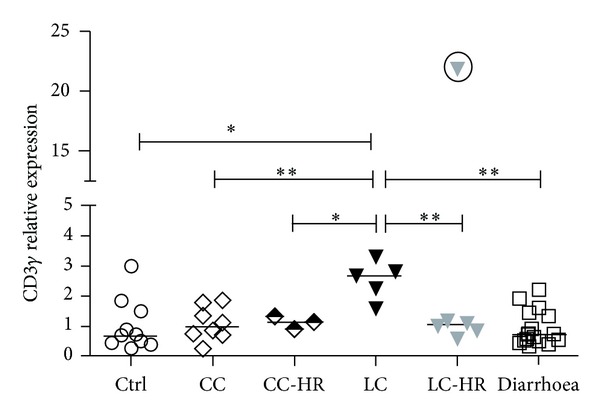
Mucosal mRNA levels of CD3*γ* in patients with collagenous colitis (CC), CC-histopathological remission (CC-HR), lymphocytic colitis (LC), LC-histopathological remission (LC-HR), noninflamed diarrhoea patients, and controls. The relative expression was calculated by the 2^(−ΔΔCt)^ method. Each symbol represents data from one individual. One symbol with the circle in LC-HR group represents a statistical outlier and was excluded from the statistical calculations. Controls, *n* = 10; CC, *n* = 8; CC-HR, *n* = 3; LC, *n* = 5; LC-HR, *n* = 6; and noninflamed diarrhoea, *n* = 17. LC patients showed statistically significant differences (^∗,∗∗^ *P* < 0.05, *P* < 0.01) compared to other groups.

We also recorded the total amount of CD3^+^ T lymphocytes in the colonic biopsies, by estimating the mucosal mRNA levels for the CD3*γ* gene. Although there was a trend towards increased relative expression of CD3 in all MC subgroups examined, the upregulation reached statistical significance only in LC patients (*P* < 0.05) compared to controls (Figure 3). LC-HR patients presented with reduced expression of CD3 gamma compared to LC patients with histologically active disease (Figure 3). 

In fact, LC patients had significantly increased CD3 gamma mRNA levels also compared to CC (*P* < 0.01), CC-HR (*P* < 0.05), and noninflamed diarrhoea patients (*P* < 0.01) (Figure 3).

## 4. Discussion

This study investigated TRECs level in the colonic mucosa of patients with CC and LC. Here we demonstrate lower levels of TRECs in the mucosa of both CC and LC patients compared to controls, suggesting that the previously observed increased numbers of T cells in the mucosa of CC and LC patients [16] are due to the expansion of local resident T cells rather than direct recruitment of recent thymic emigrants to the mucosa. This is in contrast to our previous report on increased levels of TRECs in mucosal lymphocytes from the colonic mucosa of UC patients (but not CD patients) compared to controls [17]. As that study was performed on isolated lymphocytes from resected colons, we also investigated TREC levels in colonic biopsies from 4 UC patients in the present study, and similarly to our previous observations [17] we found increased TREC levels compared to controls.

These results indicate major differences in pathogenesis between UC/CD patients and MC patients, where the latter would likely not benefit from treatment with antibodies blocking homing to the intestinal mucosa, where, for example, Natalizumab has shown good responses in CD patients [18]. This also shows clearly that it is indeed a local mucosal antigen triggering the T cell activation in MC pathology, possibly by one or several microbiota-derived antigens or by drugs that may aggravate MC in some patients [19]. 

In the absence of reliable phenotypic markers for RTE, TRECs quantification has become the method of choice to monitor newly generated T cells migrating to the periphery [9] and has been used to determine thymic export in patients with rheumatoid arthritis [20, 21], multiple sclerosis [22] and UC [17]. Reduced TREC levels have also been observed in peripheral blood lymphocytes from patients with rheumatoid arthritis [20, 21] and multiple sclerosis [22], compared to controls. However, reduced TREC levels in peripheral blood lymphocytes might very well be accompanied by increased levels in the inflamed tissue. In fact, we have previously published data showing a trend towards reduced TREC levels in peripheral blood lymphocytes from patients with ulcerative colitis [17]. 

We recently published an immunohistochemical [23] as well as a flow cytometric study [16] demonstrating elevated proportions of both LPLs and IELs expressing the Ki67^+^ phenotype, identifying proliferating cells, in both CC and LC patients. In the latter study both CD8^+^ and CD4^+^ T cells were found to harbour increased proportions of cells expressing Ki67. These studies together with the reduced TREC levels in the colonic mucosa of CC and LC patients in the present study suggest that MC patients have local expansion of resident T cells rather than migration of peripheral lymphocytes to the inflamed mucosa. Involvement of luminal agents [4] and microbes in the gut epithelium [24, 25] has previously been reported in MC pathology and our present and previous studies indicate that this initiates strong T cell responses, resulting in expansion of local T cells rather than recruitment of peripheral lymphocytes to the mucosa. Constant exposure of luminal agents to the activated T cells could lead to proinflammatory responses resulting in epithelial damage and chronic inflammation in the intestinal mucosa, as observed in MC.

As MC patients are generally of old age, we were concerned that the recorded decreased median TREC levels could be due to the older age in MC patients compared to controls. Our correlation analysis showed no significant correlation between reduced TREC levels and increasing age in the control group, consisting of a wider age span, suggesting that the observed reduced TREC levels in MC patients are indeed due to the disease itself.

Mucosal mRNA expression of the CD3*γ* gene was increased in CC and especially in LC patients compared to controls. This is in line with recent data on increased CD3^+^ T cells in both CC and LC patients [16]. Our recent data on increased proportions of CD8^+^ T cells but unaltered or reduced proportions of CD4^+^ T cells in LC patients [16] indicates that significantly increased mucosal mRNA expression of the CD3*γ* gene in LC patients stems from mucosal CD8^+^ T cells.

LC-HR patients did not demonstrate normalized TREC levels compared to LC patients, and similarly, we found no significant differences, neither in the proportion of mucosal T cells expressing Ki67 and the active/memory marker CD45RO nor mucosal mRNA and protein expression of effector T cell cytokines compared to LC patients with histopathologically active disease [16, 26], suggesting that mucosal T cell responses in LC-HR patients are as active as in LC patients.

CD3*γ* mRNA expression was not altered in noninflamed diarrhoea patients, but they presented with dramatically enhanced TREC levels compared to controls, indicating that in contrast to MC patients, the majority of the mucosal T cells of noninflamed diarrhoea patients are recently migrated from the periphery to the intestinal mucosa. This group of patient is likely heterogeneous and the cause(s) of their symptoms is unknown. The most plausible cause of diarrhoea in these patients is diarrhoea predominant irritable bowel syndrome (IBS-D). Several studies have reported low-grade mucosal inflammation and immune activation in IBS patients [27–29], including increased numbers of CD3^+^ LPLs [29], but TREC levels in IBS patients have not been assessed previously. Our recent flow cytometry data showing increased numbers of CD3^+^ LPLs in the mucosa of noninflamed diarrhoea patients, albeit still lower than in MC patients [16], is similar to the immunohistochemical findings in IBS patients by Cremon et al. [28]. Together this suggests that noninflamed diarrhoea patients represent patients with IBS-D.

Our study also indicates that the concentration of TRECs in mucosal biopsies could potentially be of future clinical value. According to our data, a patient with diarrhoea with a relative TREC expression >1.2 can be excluded from a microscopic colitis diagnosis (Figure 2).

The observations in the current study should be interpreted with caution, as it is performed on small cohorts. 

In conclusion, this study demonstrates lower levels of TRECs in the mucosa of both CC and LC patients compared to controls, suggesting local expansion of resident T lymphocytes in the inflamed mucosa of MC patients. Future investigations would try to sort CD3^+^ intraepithelial and lamina propria T cells, analyze for TREC levels, and perform multivariate analysis by considering different parameters that directly affect the TRECs measurement (age, intracellular degradation of TRECs, cell proliferation, etc.).

## Figures and Tables

**Table 1:** Demographic features of the patients and controls.

	Age (years)^a^ at the time			Disease duration	Bowel movements/day^a^	On treatment (total)	
	Sex (F/M)	of disease diagnosis	of investigation	(years)^a^		NSAIDs	PPIs
CC	7/1	61 (46–81)	63 (56–84)	4 (0–17)	5 (3–12) (*n* = 7)/ND (*n* = 1)	2 (8)	0 (8)
LC	5/0	69 (60–86)	69 (60–86)	0 (0–7)	6 (6–20) (*n* = 4)/ND (*n* = 1)	0 (5)	0 (5)
CC-HR	3/0	49 (42–59)	58 (43–63)	4 (1–9)	4 (2–4)	0 (3)	0 (3)
LC-HR	6/0	64 (23–79)	66 (23–79)	1 (0–3)	4 (3–6) (*n* = 5)/ND (*n* = 1)	0 (6)	0 (6)
NI diarrhoea	13/4	NA	58 (22–77)		4 (*n* = 1)/ND (*n* = 16)	ND	ND
UC	2/2	NA	54 (33–67)		10 (*n* = 1)/ND (*n* = 3)	ND	ND
Controls	6/4	NA	64 (42–88)			ND	ND

CC: collagenous colitis; LC: lymphocytic colitis; CC-HR: CC-histopathological remission; LC-HR: LC-histopathological remission; NI diarrhoea patients: noninflamed diarrhoea patients; NSAID: nonsteroidal anti-inflammatory drugs, and PPIs: proton pump inhibitors.

^a^Median (min–max), ND: no data, NA: not applicable.
